# Clinical TVA-based studies: a general review

**DOI:** 10.3389/fpsyg.2015.00290

**Published:** 2015-03-18

**Authors:** Thomas Habekost

**Affiliations:** Department of Psychology, University of Copenhagen, CopenhagenDenmark

**Keywords:** ADHD, alexia, assessment, attentional deficit, dyslexia, neglect, neurodegenerative disease

## Abstract

In combination with whole report and partial report tasks, the theory of visual attention (TVA) can be used to estimate individual differences in five basic attentional parameters: the visual processing speed, the storage capacity of visual short-term memory, the perceptual threshold, the efficiency of top–down selectivity, and the spatial bias of attentional weighting. TVA-based assessment has been used in about 30 studies to investigate attentional deficits in a range of neurological and psychiatric conditions: (a) neglect and simultanagnosia, (b) reading disturbances, (c) aging and neurodegenerative diseases, and most recently (d) neurodevelopmental disorders. The article introduces TVA based assessment, discusses its methodology and psychometric properties, and reviews the progress made in each of the four research fields. The empirical results demonstrate the general usefulness of TVA-based assessment for many types of clinical neuropsychological research. The method’s most important qualities are cognitive specificity and theoretical grounding, but it is also characterized by good reliability and sensitivity to minor deficits. The review concludes by pointing to promising new areas for clinical TVA-based research.

## Introduction

The theory of visual attention (TVA) is a mathematical model of visual attention that describes the process of selecting and encoding visual categorizations into short-term memory (Bundesen, 1990). The model accounts for many classical findings on visual attention from the cognitive and neurophysiological literature (see Bundesen and Habekost, 2008, 2014). Coupled with two experimental tasks, whole and partial report, TVA can also be used to estimate a set of basic attentional parameters in a given individual (Duncan et al., 1999). These parameters include visual processing speed, storage capacity of visual short-term memory, efficiency of attentional control, spatial bias of attention, and the visual perception threshold. This way, TVA-based assessment of visual attention is both grounded in basic research and provides highly specific measurements of several cognitive abilities.

Since 1999, these testing qualities have motivated widespread use of TVA-based assessment in studies of attentional deficits in neurological and psychiatric patients. The investigations fall under the general heading of clinical TVA-based studies and are the focus of the present review. About 30 such studies have been published to date, which can be grouped into four research areas: (1) neglect and simultanagnosia, (2) reading disturbances following stroke (alexia) or as a developmental disturbance (dyslexia), (3) aging and neurodegenerative diseases, and most recently (4) neuropsychiatric and neuropaediatric disorders. This article reviews the progress made in each of these research areas and discusses general trends in the empirical findings. The article also reviews methodological aspects of TVA-based assessment and looks toward promising new areas for clinical TVA-based research.

Besides clinical studies, it is important to note that TVA-based assessment has also been applied in other types of investigations that target attentional function in young healthy participants. The most important of these research lines deals with physiological or cognitive interventions designed to alter normal attentional function: transcranial magnetic stimulation (TMS; Hung et al., 2005, 2011), transcranial direct current stimulation (tDCS; Moos et al., 2012), pharmacological intervention (Finke et al., 2011; Vangkilde et al., 2012), meditation (Jensen et al., 2012), and video gaming (Wilms et al., 2013). A related line of research seeks to identify neural correlates of the TVA parameters using neuroimaging methods: functional magnetic resonance imaging (fMRI; Gillebert et al., 2012) and electroencephalography (EEG; Wiegand et al., 2014b). Studies of healthy young participants are not covered in the present review, but as suggested in Section “Future directions,” it seems promising to combine clinical TVA-based research with these intervention and neuroimaging methods.

## Methodology of TVA-Based Assessment

### The TVA Model

Before explaining the assessment method, a brief introduction to the TVA model is necessary (see also Bundesen and Habekost, 2008, for a more detailed description). According to TVA conscious recognition of a visual object corresponds to encoding one or more of the object’s properties into a visual short-term memory store. The memory store only has room for very few objects, typically 3 or 4 in young healthy individuals, and its capacity (the *K* parameter in TVA) varies individually. The encoding process that leads up to conscious recognition takes the form of a competitive race between the objects in the visual field. TVA assumes that the visual system processes all objects in the visual field independently and in parallel, but not equally fast. The processing rate of a given object determines its probability of winning the race and become encoded into visual short-term memory, given that the store is not already filled up by other objects. The sum of the processing rates for all objects in the visual field equals the total processing speed of the visual system (under the given stimulus conditions) and is represented by parameter *C*. The processing rate for each individual object reflects the proportion of the total processing capacity that has been allocated to the object (its attentional weight). The computation of attentional weights is also modeled in TVA, and comparisons between the weights of different objects provide the basis of two additional parameters. The attentional weight of a target versus a distractor object is a measure of the efficiency of top–down control of attention (parameter α). Making an alternative comparison, the attentional weights of objects in different parts of the visual field (e.g., left vs. right) provide a measure of spatial attentional bias (parameter *w*_index_). The fifth parameter of the TVA model, *t*_0_, represents the time at which the processing race starts and visual objects begin to have an above-zero probability of being recognized. This implies that *t*_0_ is measure of the lower threshold for visual perception.

The TVA model is formulated mathematically (see Bundesen and Habekost, 2008, for details) and can therefore provide exact predictions for attentional performance under specific experimental conditions. Bundesen (1990) used this quantitative precision to model a wide range of classical findings in the literature on normal visual attention. The empirical account by Bundesen (1990) covered many experimental paradigms such as whole report, partial report, cued detection, single stimulus recognition, and visual search. It has also been shown that the TVA model can explain many of the attentional effects observed at the single-cell processing level, as reflected in firing rates of individual neurons (Bundesen et al., 2005).

### TVA-Based Assessment: General Method

**FIGURE 1:**
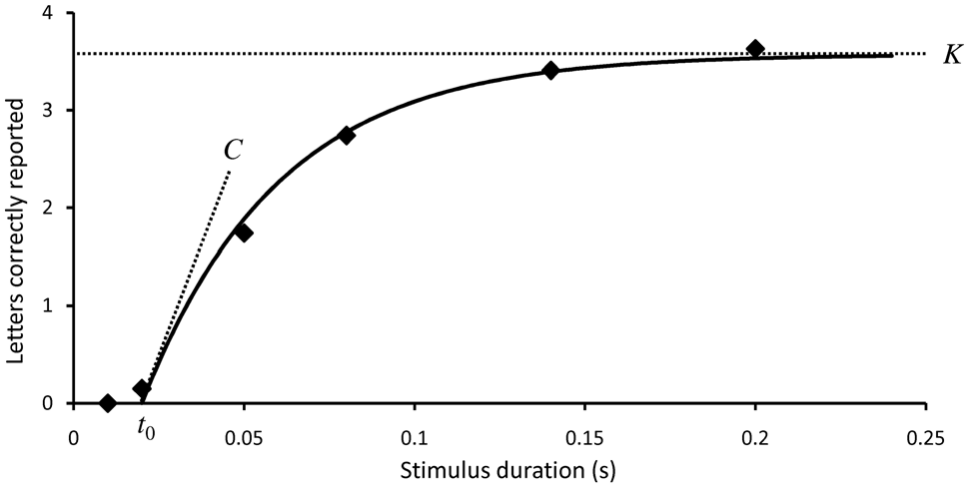
**Typical performance in a whole report experiment.** The mean number of correct reports (the score) is shown as a function of exposure time. Solid curves represent maximum likelihood fits to the observations based on TVA analysis. The three TVA parameters that can be derived from whole report data are marked out: the perceptual threshold *t*_0_, the visual processing speed *C*, and the storage capacity of visual short-term memory *K*.

TVA can account for findings from many different attentional paradigms, but individual values of the model’s five parameters can be estimated most directly from performance on two specific tasks, whole report and partial report. Indeed, all studies with TVA based patient assessment use some version of whole report and/or partial report to estimate some or all of the parameters specified in the TVA model. In whole report one or more visually simple objects (typically letters) are flashed on a computer screen for a well-defined time period (the exposure time). The stimuli are typically followed by pattern masks, which erase the visual afterimage and precisely control the time the stimuli are available for processing. In other cases the stimuli are followed by a blank screen, which extends the effective exposure duration (the prolongation can be approximated by a constant, parameter μ) and thereby makes the task easier, which is relevant in many clinical contexts. The participant is instructed to report the identity of as many objects as possible, but refrain from guessing. Reporting must neither be too liberal or too conservative, which is usually operationalized as an error rate between 10 and 20% and controlled by feedback to the participant during testing. Some participants (e.g., young children or elderly persons) may find it difficult to comply with this well-defined accuracy level; in such cases forced-choice reporting can be employed coupled with correction for guessing in the data analysis. It is also recommended to precede testing with a short practice period (e.g., 30–40 trials) where participants can be familiarized with the task. When testing is repeated a sufficient number of trials, and with a selection of exposure times that avoids floor and ceiling effects, a systematic pattern of whole report performance then emerges (see **Figure 1**).

Below an individually variable exposure duration *t*_0_, the number of correctly reported letters is zero. *t*_0_ is therefore a measure of the visual perception threshold for the stimuli. As the exposure duration is increased above the threshold the score increases monotonically, though with negative acceleration. The slope of the curve at its steepest point, *t*= *t*_0_, is a measure of the total visual processing speed *C*. At higher exposure durations the performance curve gradually levels off to approach an asymptote, which represents the maximum storage capacity of visual short-term memory, *K*. *K* is classically assumed to represent a weighted average of two maximum values (Shibuya and Bundesen, 1988). For example, storage capacity might alternate between 3 and 4 elements with a probability of occurrence at 40 and 60%, respectively. Recent developments of TVA analysis, however, assume a more broad distribution of *K* (see Reliability and other Psychometric Properties). If stimuli are simultaneously shown in both visual fields the spatial balance of attentional weights, *w*_index_, can also be estimated from whole report data. *w*_index_ range from 0 to 1; symmetrical attentional weighting corresponds to a *w*_index_ value of 0.5 and the degree of lateral bias to either the left or right side can be measured by the deviation of *w*_index_ from this intermediate value.

In the partial report task stimuli of two different types (typically distinguished by color) are presented. The task is to report only stimuli of one type (targets) and ignore the other stimuli (distracters). The performance reduction with distracters, compared to the situation when only targets are presented, provides a measure of the efficiency of top–down selectivity α. More specifically, α is defined as the ratio of attentional weights between a target and a distracter. An α value of 0 implies perfect selection, whereas a value of 1 implies no selectivity between targets and distracters.

### Variants of TVA-Based Assessment

**FIGURE 2:**
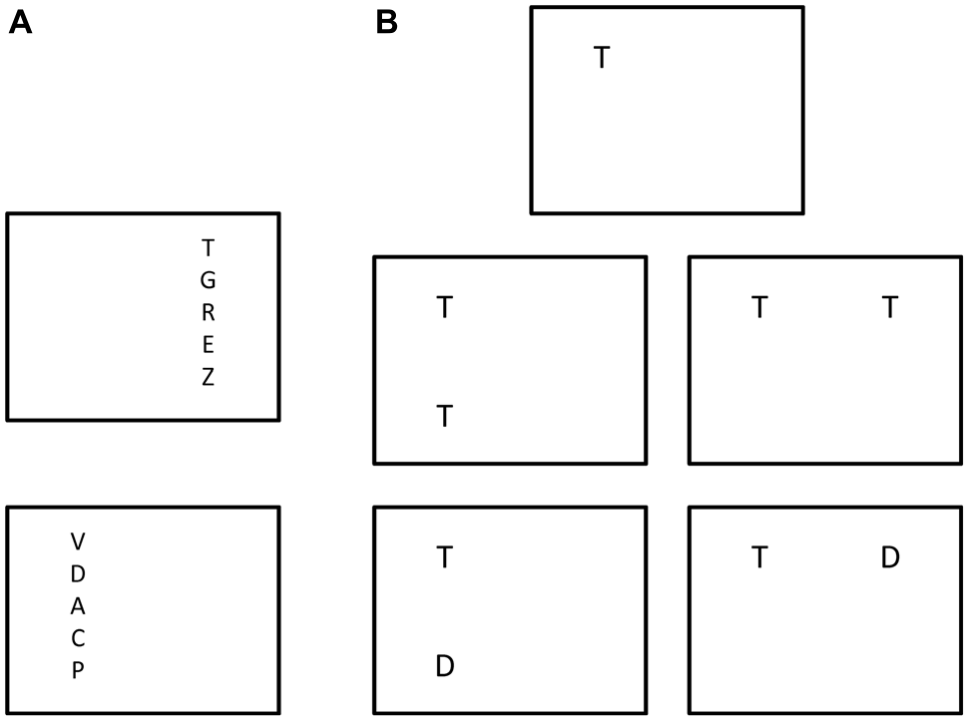
**The original version of TVA based assessment devised by Duncan et al. (1999). (A)** Different trial types of the whole report experiment. **(B)** Different trial types of the partial-report experiment with targets (marked as “T”) and distracters (marked as “D”).

The basic method of TVA based patient assessment was developed by Duncan et al. (1999), who used it to study patients with neglect following stroke in the right hemisphere (see Neglect and Related Conditions for the main findings of this study). Duncan et al. used a whole report task where five letters are displayed in a vertical column either to the left or right of fixation, shown at three individually adapted exposure times (see **Figure 2A**). In half of the conditions the stimuli are post-masked, in the other half they are followed by a blank screen. This way separate estimates of *C*, *K*, and *t*_0_ can be obtained in each hemifield. Duncan et al. also used a partial report experiment, where either one or two stimuli (targets or distracters, defined by color) are shown at four possible locations around fixation, at one individually adapted, post-masked exposure duration (see **Figure 2B**). This partial report paradigm allows for estimation of α and provides an indirect measure of sensory effectiveness by the parameter *A* (in which the effects of *t*_0_ and *C* are not separated) for each of the four display positions. An estimate of *w*_index_ can also be derived from this paradigm due to the inclusion of bilateral target displays.

**FIGURE 3:**
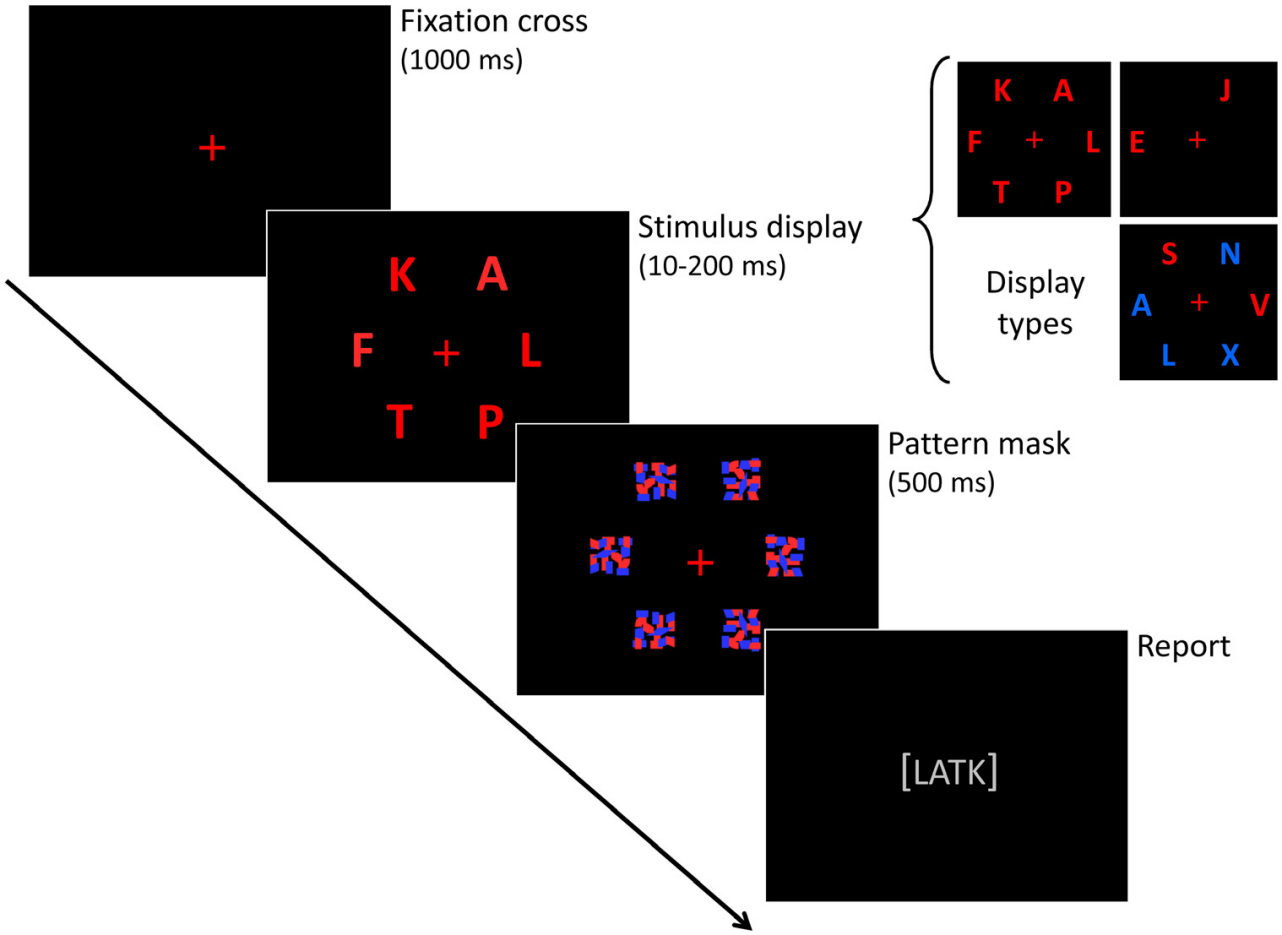
**The CombiTVA paradigm devised by Vangkilde et al. (2011).** The outline of a single trial is shown, together with the three trial types.

The experimental design used by Duncan et al. (1999) has been adopted in many later studies, especially those carried out by German research groups (e.g., on neurodegenerative diseases) and in this way established as a standard paradigm for TVA based assessment. Other studies have altered the details of the experimental design considerably, either to optimize testing for a specific clinical group or to elucidate a particular research hypothesis. For example, Peers et al. (2005) used displays with just one centrally located stimulus to estimate visual processing speed. Habekost and Rostrup (2006, 2007) used circular displays and included a version of partial report with multiple targets and distracters. The stimuli themselves have also been varied, both with regards to letter appearance (color, font, size) and in some cases by including other stimulus types (e.g., faces: Peers et al., 2005; digits: Starrfelt et al., 2009; short words: Habekost et al., 2014a). In recent years a second standard paradigm for TVA based assessment has emerged, the so-called CombiTVA paradigm developed by Vangkilde et al. (2011). The CombiTVA paradigm uses six display stimuli and intermixes whole and partial report trials to better constrain the total set of parameter estimates (see **Figure 3**). The CombiTVA has already been used quite widely in studies that together number many hundred participants (e.g., Dyrholm et al., 2011; McAvinue et al., 2012b; Habekost et al., 2014b).

### Developments in Data Analysis

The procedure used by Duncan et al. (1999) to analyze their data was specific to the experimental design in this study. The analysis method was mathematically generalized by Kyllingsbæk (2006), who also provided an easy-to-use software package for automated analysis of many types of whole and partial report data. This fitting program has been used for a large part of the studies described in the present article. Dyrholm et al. (2011) further developed the model fitting procedure. Besides presenting a more analytically efficient procedure for parameter estimation, they documented a significant trial-by-trial variability in a large data set from 347 healthy participants, which was not captured by the standard TVA data modeling. Further, systematic biases were demonstrated in the standard estimation of the *K* and *t*_0_ parameters: *K* was typically overestimated by at least half an item, whereas *t*_0_ was underestimated by about 2 ms. The modified analysis procedure of Dyrholm et al. enables more robust fitting of the *K* parameter by assuming that the parameter follows a broader distribution (from trial to trial) rather than varying between just two maximum values. In addition, the *t*_0_ parameter is allowed to vary from trial to trial following a Gaussian distribution to counter underestimation of the perceptual threshold due to a few “lucky guesses” during testing at short exposure durations. A new fitting parameter, the probability of an attentional “lapse” during testing (i.e., no report despite a long exposure duration) was also proposed by Dyrholm et al. and has been used in a recent study of children with ADHD (McAvinue et al., 2012a).

### Reliability and other Psychometric Properties

The psychometric properties of TVA-based assessment, especially the reliability of the tests, have been investigated in several ways. In two studies of stroke patients, Habekost and Bundesen (2003) and Habekost and Rostrup (2006) used bootstrap statistics to estimate the measurement error related to each of the TVA parameters (see Neglect and Related Conditions for further descriptions of these studies). In brief, bootstrap analysis produces an estimate of the variability inherent in a set of observed data (e.g., 324 observations in a whole report experiment) and provides a statistical confidence interval for each parameter estimate that is based on these data (Efron and Tibshirani, 1998). The bootstrap analysis showed generally low measurement error for most parameter estimates, especially the *K* parameter. Notably, the estimates of the α parameter were found to be less reliable than the others. The analysis was also useful for testing whether parameter estimates were significantly different from each other in the left or right hemifield, a major focus of these two studies.

The bootstrap method was used in a different way by Finke et al. (2005), who studied 35 young healthy participants by whole and partial report tasks (using the standard paradigms of Duncan et al., 1999). Finke et al. compared estimates based on the full data set (672 trials) vs. subsets of the data (i.e., the first 384, 288, or 192 trials) for each participant to investigate how test length affected the reliability of the parameter estimates. The bootstrap analysis indicated high internal reliability even for the shortest versions of the data set, with α as the least reliable parameter. This analysis was supplemented by computations of intra-parameter correlations between estimates based on the full or partial versions of the data set. This analysis showed very high stability of *C* and *K* (*r* > 0.90) even after just 192 trials, whereas α and *w*_index_ obtained sufficient reliability (*r*≥ 0.75) only with 288 or more trials. Finke et al. also looked at the intercorrelations between different TVA parameters and found these to be generally small and non-significant, apart from a moderately strong relation between *C* and *K* (*r* = 0.40). This was taken as evidence of good functional specificity in the test’s measurement. The specificity of the TVA parameters was further supported by the fact that each of them correlated well with clinical tests addressing corresponding cognitive functions.

The reliability of TVA based assessment was investigated more generally by Habekost et al. (2014b) in a sample of 68 young healthy participants, who were tested three times (1 week interspaced) with the CombiTVA paradigm (432 trials in total). The data modeling was carried out in accordance with the modified procedure of Dyrholm et al. (2011). The results were compared to another widely used test of attention, the Attentional Network Test (ANT; Fan et al., 2002), which is theoretically based in Posner and Petersen (1990) anatomical network model of attention. In line with the previous bootstrap investigations, Habekost et al. (2014b) found high or very high internal reliability (*r* = 0.90 or higher) for all five TVA parameters by a split-half analysis of the data. The very high internal reliability held up for *K* and *w*_index_ in a shortened version of the CombiTVA (216 trials), whereas the other three parameters still showed good, but not high reliability under these circumstances. The study also provided the first systematic investigation of the test–retest reliability of TVA based assessment. *K* and *w*_index_ showed good retest reliability (*r* > 0.80) throughout the repeated testing, whereas *C*, *t*_0_, and α showed only moderate reliability (*r* around 0.60) from the first to the second testing session, which, however, increased to *r* = 0.75–0.85 between the second and the third testing. Both the internal and test–retest reliability of the CombiTVA was consistently better than that of the ANT test, also when taking test length into account. Further, the results replicated Finke et al.’s (2005) finding of small and non-significant intercorrelations between the different TVA parameters, except for *K* and *C*, which in this study were highly correlated (*r* = 0.72). TVA parameters were generally not related to the ANT measures either, apart from a moderate correlation between α and the ANT’s executive network score. Finally, the study showed significant practice effects over the course of the three testings, especially for the *C* and α parameters. This makes it clear that performance on TVA based assessment can only be meaningfully compared between participants with equal levels of practice with the task.

### Section Summary

A full evaluation of the strengths and weaknesses of TVA-based assessment must include studies that have employed the method in clinical populations. These studies are to be presented in the next four sections, but the studies described in this section still allow for a number of preliminary conclusions. As a test of visual attention in healthy participants TVA-based assessment has a number of important strengths. One of the most important is the method’s theoretical grounding, the fact that it is derived from a general model of visual attention that accounts for a large part of the basic research within the field. This implies that the parameters measured are not narrowly bound to particular testing tasks, but represent more general aspects of attentional function. The ANT test of Fan et al. (2002) has a similar status by being grounded in Posner’s anatomical network model of attention, which can also explain many findings in the attentional literature. The ANT, however, measures other, more response-related aspects of attention and produces less reliable test results than TVA-based assessment.

Another main strength of TVA-based assessment is the method’s specificity: five different aspects of attention are being measured separately. Correlations between the different TVA parameters are generally low, with the exception of *C* and *K*, which may raise some concern about the empirical separability of these two parameters. The specificity of the assessment is also evident in another way: unlike most other tests of attention, whole and partial report tasks do not involve reaction-time measures, which means that motor processes do not influence performance significantly. For this reason the test results specifically reflect the efficiency of attentional processes within the visual system. TVA-based assessment also has good reliability, both as measured within a single test session and across re-testings. Practice effects, however, need to be controlled for when comparing performance between individuals, and estimation of some parameters (e.g., α) is less reliable than others.

From a practical consideration, it is useful that the measurement of all parameters is carried out in one integrated test setup with simple instructions and minor response requirements. The fact that the experiments can be tailored to different theoretical interests and test populations (e.g., by varying the stimulus types and display arrangement) provides flexibility for many types of investigations. The test produces reliable results from a few hundred trial repetitions, which implies that a minimum of about half an hour’s testing is necessary. This time frame is compatible with many clinical examinations, but can still be an obstacle in some situations (e.g., bed-side testing). This leads us to the clinical studies.

## Neglect and Simultanagnosia

Neglect is a severe disturbance of attention that often follows unilateral stroke, especially after right-side lesions. Neglect can be defined as the failure to report, respond, or orient to stimuli in the contralesional side, when this failure cannot be attributed to either sensory or motor impairments (Heilman et al., 2003). Neglect is probably the most widely studied attentional syndrome in the neuropsychological literature and provided a natural starting point for clinical TVA-based studies when the field was opened up by Duncan et al. (1999). The primary aim of Duncan et al. was to use the cognitive specificity of TVA-based assessment to characterize different components of the neglect syndrome. This general idea of cognitive deficit analysis has inspired many of the other studies described in this review. Subsequent TVA-based investigations of neglect and similar conditions have followed up on the initial study of Duncan et al. in various ways. For example, studies have used TVA-based assessment to clarify the lesion anatomy underlying different attentional deficits or to characterize subclinical manifestations of the neglect syndrome.

Another classic neuropsychological syndrome, simultanagnosia, has also been investigated by TVA-based testing. Simultanagnosia is an even more severe attentional disturbance than neglect and characterized by an extreme reduction in the ability to perceive scenes and multiple objects at the same time. Simutanagnosia typically occurs after bilateral parietal lesions (dorsal simultanagnosia) in which case it is often associated with difficulties in visually guided reaching and eye movement control (Balint’s syndrome). Also in the studies of simultanagnosia, a main aim has been to specify different functional deficits and thereby address theoretical hypotheses about the core deficits of the syndrome.

### Neglect and Related Conditions

#### Duncan et al. (1999): Neglect

Duncan et al. (1999) studied nine patients with visual neglect after strokes in the right hemisphere. The lesions centered on the right parietal cortex, but adjacent structures were also affected to some extent. Compared to an age-matched control group Duncan et al. (1999) found that the patients had general reductions in processing capacity: *K* was abnormally low in both hemifields. *C* was also reduced bilaterally, most pronounced in the left hemifield. Given that neglect is traditionally associated with lateralized deficits, this was an interesting finding, and one that corresponds well with later understandings of the syndrome that emphasize non-lateralized deficits (e.g., Husain and Rorden, 2003). In addition, a significant bias of attentional weights (as measured by *w*_index_) for stimuli in the right visual field was found, consistent with the defining symptoms of neglect. Surprisingly, top–down control of selectivity was found to be intact, even in the left hemifield: α values were normal in both sides. Although the findings on α would later be questioned on grounds of low reliability for this parameter, Duncan et al. (1999) did provide a first demonstration of how TVA-based assessment can distinguish between impaired and preserved aspects of visual attention, inspiring many later studies.

#### Habekost and Bundesen (2003): Subclinical Neglect

Habekost and Bundesen (2003) presented the first TVA-based study of brain damage outside the parietal cortex. The patient investigated in this single case study had damage to the right basal ganglia and overlying frontal cortex, but no neglect or other attentional disturbances as measured by standard clinical testing. However, she did have subjective complaints about poor apprehension of events in the left side. In this sense, the study can be viewed as the first investigation of subclinical attention disturbances by TVA based testing. The patient’s subjective complaints were confirmed by the TVA-based testing: in the partial report experiment, where stimuli were presented very near the perception threshold (40 ms), performance was clearly better in the right hemifield (reflected in both *A* and *w*_index_ values). This pattern differed markedly from the control group. In addition the patient’s *K* parameter was significantly reduced in both sides, and the visual perception thresholds were elevated, particularly in the left side. Given that neither of these deficits was evident from standard testing, the study showed the sensitivity of TVA based assessment to subtle attentional deficits.

#### Habekost and Rostrup (2006, 2007): Right Hemisphere Stroke

Habekost and Rostrup (2006) followed up on this case study by a group investigation of 26 patients with right hemisphere strokes. The strokes varied widely in size, but in most cases centered on the basal ganglia and overlying frontal cortex, with variable additional involvement of the temporal and parietal cortices. Four patients had small lesions that were restricted to the right thalamic area. As in the study of Habekost and Bundesen (2003), most patients had minor or no deficits on standard tests of neglect. Lateralized abnormalities in the TVA-based testing were, however, widespread in the patient group: visual processing speed was markedly lower in the left than the right hemifield for almost all patients in the group. In addition, when presented with bilateral displays, patients with large strokes showed an abnormally biased attentional weighting toward stimuli in the right hemifield (*w*_index_). This pattern was not found after small lesions, however, with one important exception: a patient with thalamic damage involving the pulvinar nucleus showed a similarly biased attentional weighting. The finding corresponds well with an anatomical interpretation of the TVA model that proposes a central role for the pulvinar in the computation of attentional weights (Bundesen et al., 2005).

In a supplementary analysis of data from the same study, Habekost and Rostrup (2007) found that both visual short-term memory and visual processing speed in the ipsilesional field were normal for most patients, in spite of their large lesions. This implies that lesions in a large region of the right hemisphere, inclucing the putamen, insula, and inferior frontal cortex, do not lead to general deficits in either *C* or *K*. Deficits in *K* did, however, occur in patients with severe leukoaraiosis or lesions extending deep into white matter, suggesting a vital role for white matter connectivity for visual short-term memory function.

#### Kraft et al. (2015): Thalamic Stroke

Following up on the case investigation on thalamic damage by Habekost and Rostrup (2006), Kraft et al. (2015) presented a larger group study of patients with thalamic lesions. Sixteen patients with focal damage in different subregions of the thalamus (either left or right side) were tested by the whole and partial report tasks of Duncan et al. (1999). Their lesions were examined by structural magnetic resonance imaging and mapped in standard stereotactic space. Compared to an age-matched control group the patients were on average mildly impaired in terms of *C* and *K* values. Lateral thalamic lesions were related to deficits in visual processing speed, whereas medial thalamic lesions were associated with asymmetrical attentional weighting, as measured by *w*_index_. This implies that patients with lesions outside the traditionally defined visual areas of the thalamus showed deficits on the TVA parameters. The performance of one patient with pulvinar damage replicated the finding of Habekost and Rostrup (2006), a spatial bias to the ipsilesional field, but this time shown after a left-side lesion.

#### Peers et al. (2005): Parietal vs. Frontal Strokes

Peers et al. (2005) also investigated the effects of unilateral stroke. This study included 25 patients with lesions restricted to the parietal cortex (13 patients) or the frontal cortex (12 patients), either in the left or right hemisphere. In one experiment the patients’ visual processing speed was tested by means of a single, post-masked stimulus (either a letter or a face). The results showed that patients with parietal, but not frontal, lesions had significantly reduced *C* values for both letters and faces. In a second experiment six target letters were displayed for a relatively long time (200 ms) without masking to obtain a rough estimate of the maximum number of letters that could be perceived (i.e, *K*). Again, patients with parietal lesions were on average impaired, but patients with frontal damage did not differ significantly from the control group. The results from both experiments thus indicated that deficits in processing capacity (*C* or the derived *K* measure) were selectively associated with parietal lesions. Deficits related to attentional weighting, however, showed quite a different pattern: both α and *w*_index_ were related to the volume of lesion, not its location. The finding on *w*_index_ was in line with the results of Habekost and Rostrup (2006), who also found that large lesions were related to unbalanced attentional weighting.

#### Bublak et al. (2005): Parietal vs. Frontal Stroke

Bublak et al. (2005) conducted a similar comparison between the effects of lesions in the parietal and frontal lobe. The study was based on two case investigations, where a patient with a large stroke involving the right inferior parietal lobe was compared to a patient with a circumscribed lesion in the right superior frontal lobe. The standard partial report paradigm of Duncan et al. (1999) was used. The patient with inferior parietal damage showed similar deficits to the patients in the study of Duncan et al.: rightward spatial bias (*w*_index_), reduced sensory effectiveness in the left visual field (parameter *A*), and preserved top–down control (α; Bublak et al., 2005 however, noted that this parameter could be reliably measured only in the right visual field). Interestingly, the other patient with superior frontal damage showed a complementary deficit in parameter α, but not in the other parameters, forming a double dissociation. A whole report test of the patient with parietal damage showed additional bilateral deficits in *C* and *K*, consistent with the findings of Duncan et al. Because the study only involved two patients, Bublak et al. could not conduct a systematic lesion analysis like Peers et al. (2005). Instead, the main contribution of the study was to demonstrate that short versions of the tests (30–40 min) were sufficient to demonstrate a double dissociation of deficits between two stroke patients, supporting both the specificity and clinical usability of TVA-based assessment.

#### Finke et al. (2012): Phasic Alerting of Neglect Patients

Finke et al. (2012) assessed whether phasic alerting can influence the deficits in processing capacity and attentional weighting that are characteristic of neglect patients. The study was inspired by Robertson et al. (1998) who showed that phasic alerting may alleviate neglect symptoms. Six patients with neglect following right temporo-parietal lesions were included in the study. Finke et al. (2012) used a simplified version of the second paradigm of Duncan et al. (1999; see **Figure 2B**) in which only targets were shown (i.e., whole report) and compared performance with or without pre-alerting by a visual cue. The time course of the alerting effect was investigated by varying the SOA between alerting cue and stimulus display (80, 200, or 650 ms). Finke et al. (2012) found that phasic alerting normalized the spatial imbalance of attentional weights (*w*_index_) in the control (no-cue) condition, but only for the two shortest SOAs: when 650 ms elapsed between the alerting cue and the display, the patients fell back to the usual rightward bias in *w*_index_. The effect of visual alerting on attentional weighting was thus fast evolving, but also short-lasting. The cue also increased sensory effectiveness (as measured by *A* values) mainly in the right visual field. This effect was more stable over time and evident at all SOAs.

### Simultanagnosia

#### Duncan et al. (2003): Dorsal Simultanagnosia

In line with its name, simultanagnosia is classically conceived as a failure to perceive multiple objects at the same time (Kinsbourne and Warrington, 1962). Duncan et al. (2003), however, wanted to test whether the basic deficit lies in visual processing speed rather than in the ability to perceive multiple objects simultaneously. The two types of deficits correspond closely to TVA’s distinction between *C* and *K* values (e.g., in a strong version of the classical interpretation, *K* should be only one for simultanagnosic patients). Duncan et al. (2003) tested a patient with dorsal simultanagnosia in two variations of whole report with multiple stimuli and in both cases found very severe reductions of *C*, whereas the deficit in *K* was more modest. In the third experiment only a single letter was shown at fixation. Even with no other objects competing for attention, the patient’s performance was still very low. Thus the study pointed to a deficit in processing speed, rather than simultaneous perception, as central to the condition, at least for this patient. A second patient with ventral simultanagnosia (left occipito-temporal lesion) was also tested in this study. However this condition is quite different from dorsal simultanagnosia and will be reviewed in the section on alexia (see Alexia; see also Gerlach et al., 2005, for an application of TVA-based assessment to rule out simultanagnosia in a neuropsychological case study).

#### Finke et al. (2007): Simultanagnosia and Huntington’s Disease

Simultanagnosia is traditionally associated with bilateral stroke, but Finke et al. (2007) showed how it can also occur in a neurodegenerative disturbance, Huntington’s Disease. The study was a follow-up to the first TVA-based study on Huntington’s Disease (Finke et al., 2006), which is described in Section “Neurodegenerative Diseases.” Ten Huntington’s patients were tested with tasks that required perception of multiple overlapping figures under free viewing conditions, and the number of errors was correlated to performance on a whole report experiment. Finke et al. (2007) found that a deficit in the overlapping figures test was significantly correlated with low visual processing speed (*C* values), but not VSTM capacity (*K* values). Thus the findings paralleled the study of Duncan et al. (2003), who also found that simultanagnosia is primarily related to deficits in visual processing speed.

### Section Summary

TVA-based assessment has proven to be a relevant tool for studying neglect, simultanagnosia, and related conditions. The testing is clearly sensitive to central aspects of these neuropsychological conditions, also for patients with milder deficits, and the findings are consistent across studies. Especially the specificity of the assessment method has proven valuable to disentangle different components of neglect and simultanagnosia. This deficit analysis relates directly to fundamental theoretical discussions about the core characteristics of the two syndromes. The specificity of the assessment method has also proven useful for relating deficit patterns to their underlying lesion anatomy. For example, spatial biases in attention have been found after two very different types of lesions: large unilateral strokes or focal damage to the pulvinar nucleus. Finally, the study of Finke et al. (2012) indicates that the specificity of TVA-based assessment can also be useful to chart the efficiency of rehabilitation procedures, a theme that is revisited in Section “Future Directions.”

## Reading Impairments: Alexia and Dyslexia

TVA-based assessment typically includes letters as test stimuli, which makes it natural to use the method for examining reading impairments. The main focus in this line of TVA-based research is on the basic visual efficiency of letter processing, represented by the two capacity parameters *K* and *C*. Two general types of disturbances have been investigated: alexia and dyslexia. Alexia, a selective deficit in reading ability after brain damage, typically occurs after stroke in the posterior left hemisphere (see Leff and Starrfelt, 2014, for a general overview of alexia research). In milder cases the reading disability may simply be explained by a visual field cut (e.g., hemianopia). Brain damage can, however, also affect perception of letters and words at more central processing levels. The main example of this is the classical neuropsychological syndrome of pure alexia. Pure alexia is characterized by a severe inability to read fluently, which is reflected in the so-called letter-by-letter reading pattern. Letter-by-letter reading is evident from naming tasks of single words, where reaction times for patients with pure alexia increase linearly (and strongly) with the length of the presented word. The reading deficit occurs in the absence of other problems in writing or language processes, hence the term “pure.” A long-standing discussion concerns the nature of the basic cognitive deficit that produces the letter-by-letter reading pattern (see Alexia).

The other main line of TVA-based studies on reading disturbances focuses on dyslexia. Like in alexia, the defining symptom of dyslexia is reading difficulties, but dyslexia is a developmental condition rather than a neurological one and associated with less severe reading impairments than alexia. The dyslexia studies have also used TVA-based assessment to specify basic deficits in letter processing, in order to address theoretical hypotheses about the underlying causes of the reading problem.

### Alexia

Apart from the study of Habekost and Starrfelt (2006) described at the end of this section, TVA-based studies of reading problems after brain damage have focused on pure alexia. Several theoretical hypotheses about the basic deficit of pure alexia have been proposed, which roughly fall into two categories: (1) domain-specific theories, which suggest that pure alexia is related to a deficit in recognizing visual word or letter forms (Warrington and Shallice, 1980; Cohen et al., 2003), or (2) general visual accounts, which propose that the reading problem is caused by a general deficit in visual perception (Behrmann et al., 1998) for example an impairment in simultaneous perception (Farah, 2004). The different predictions of these models can be tested directly by TVA-based assessment. For example, a letter- or word-specific account would predict severe reductions in visual processing capacity (*C* and *K* values) for orthographic stimuli, but not other stimulus types. On the other hand, a primary deficit in simultaneous perception should lead to reduced *K* values for all types of visual stimuli, whereas visual processing speed for singly presented objects could be normal.

#### Duncan et al. (2003): Ventral Simultanagnosia

The first to investigate these issues was Duncan et al. (2003) who studied a single patient with ventral simultanagnosia (i.e., pure alexia). In a whole report task with multiple letters the patient showed severe reductions of visual processing speed for letters, whereas the reduction in *K* was more modest. An attentional bias toward left-side stimuli (shown in the *w*_index_) was also shown, using the partial report design of Duncan et al. (1999). This initial investigation of pure alexia thus suggested a primary deficit in visual processing speed rather than simultaneous perception, accompanied by an attentional bias toward ipsilesional stimuli, as would be expected following a large unilateral lesion (cf. Neglect and Related Conditions).

#### Starrfelt et al. (2009, 2010): Pure Alexia

In five case studies Starrfelt et al. (2009, 2010) investigated the stimulus selectivity of pure alexia by comparing performance with letters versus digits. The testing was conducted in single-stimulus recognition experiments (targeting the visual processing speed for each stimulus type) as well as by whole report of multiple items (testing the hypothesis of a deficit in simultaneous perception). The findings were very consistent across all five patients: both *C* and *K* were markedly reduced, and to the same extent for letters and digits. Starrfelt et al. (2009) concluded that these general deficits in visual “speed and span” provide a plausible explanation for the patients’ problems with word reading. The hypothesis of a close relation between perception of individual letters and reading of whole words, however, still awaited direct investigation.

#### Habekost et al. (2014a): Pure Alexia

Habekost et al. (2014a) therefore extended the investigations to include single-stimulus recognition of three-letter words and compared this task to performance with individual letters. Vocal naming speed for both types of stimuli was also tested to assess whether the main difficulty was at the level of perceptual or response-related processes. Four patients with pure alexia were investigated, one with a relatively mild reading impairment, the others with more severe symptoms. The healthy control participants showed a clear word superiority effect: consistently better visual recognition of words than letters. In contrast, none of the four patients showed this pattern; two patients had approximately equal recognition of letters and words, whereas the other two showed a significant “word inferiority” pattern. In the vocal naming task the word inferiority pattern was even more evident for all four patients. Besides these intra-individual differences between letters and words, all patients had clearly reduced recognition accuracy for both stimulus types compared to controls, replicating the previous findings of low visual processing speed in patients with pure alexia. There was, however, one interesting exception to this general pattern: patient SH showed normal recognition of single letters, which represented a significant dissociation from his slowed vocal naming of the same stimuli, as well as his highly deficient perception and naming of words. This may be the first clear demonstration of a pure alexia patient with intact letter recognition abilities, a finding of direct relevance to the hypothesis that the basic deficit in pure alexia is in recognition of individual letters (e.g., Behrmann and Shallice, 1995).

#### Habekost and Starrfelt (2006): Quadrant-Amblyopia

Habekost and Starrfelt (2006) investigated a more subtle reading problem than pure alexia, in a patient with a condition they labeled “quadrant-amblyopia.” Following a stroke in the posterior left hemisphere, the patient’s ability to read fluently was reduced, even though other language functions were intact. The reading pattern was similar to the well-known syndrome of hemianopic alexia, but the patient did not show visual field cuts on perimetric assessment. Other known neuropsychological causes for reading impairment were also ruled out. However, TVA-based testing by whole report showed severe deficits in the visual processing speed of letters to the right of fixation (and in the upper right quadrant) whereas processing of letters was normal in other parts of the visual field. This spatially selective amblyopia (“foggy vision”) provided a plausible explanation for the patient’s mild reading problem.

### Dyslexia

Similar to alexia research, several theories of dyslexia claim that the reading disturbance is caused by impairments in specific visual processes. The suggested hypotheses include a deficit in simultaneous perception (Bosse et al., 2007), a left “mini-neglect” (Hari et al., 2001), or slower processing of individual letters (Dubois et al., 2010). This corresponds to deficits in the *K*, *w*_index_, and *C* parameters, respectively, and the hypotheses are therefore directly testable by TVA-based assessment.

#### Dubois et al. (2010): Childhood Dyslexia

The first TVA-based study of children with dyslexia was reported by Dubois et al. (2010), who studied two 9-years old children by whole report of 1, 3, or 5 letters. Dubois et al. (2010) found significant deficits in both visual processing speed and storage capacity of visual short-term memory for one of the children, whereas the other child only showed a borderline significant reduction in *C*. In both children a non-significant tendency for an attentional bias (*w*_index_) toward left-side stimuli was also noted.

#### Bogon et al. (2014): Childhood Dyslexia

Bogon et al. (2014) followed up with a group study on 12 dyslexic children (mean age 10 years) using the whole and partial report designs of Duncan et al. (1999). Bogon et al. (2014) found that *K* and *C* were on average significantly reduced, whereas α and *w*_index_ did not differ significantly from the control group. Interestingly, the *K* values of the dyslexic children correlated significantly with reading performance. This, however, contrasts with a study of normally developing children by Lobier et al. (2013), who instead found that differences in visual processing speed predict text reading speed.

#### Stenneken et al. (2011): Adult Dyslexia

In a study of 23 high-functioning young adults with dyslexia Stenneken et al. (2011) also found deficits in visual processing speed, but in this study *K* values were on average at normal levels. In addition, Stenneken et al. (2011) found that *w*_index_ values correlated with the severity of the dyslexia, even though this parameter was on average normal in the group.

### Section Summary

Research on two types of reading disturbances have been reviewed in this section: alexia and dyslexia.

Theory of visual attention-based studies of pure alexia have consistently shown severe reductions of visual processing capacity, reflected in both *C* and *K* values. The reductions have been demonstrated for individual letters as well as digits, indicating a general visual deficit. Further, the processing deficit seems to be exacerbated in case of word stimuli. This way, TVA-based testing has proven useful in addressing some of the main theoretical hypotheses within this field. However, the investigations have yet to achieve their primary aim, which is to isolate the basic processing deficit in pure alexia (if one such exists: see Starrfelt and Shallice, 2014, for a discussion of current challenges in alexia research). Instead of one deficit the results point to a set of associated impairments, each of which may cause the reading impairment.

The findings on dyslexia are similar to pure alexia, but reflect the milder nature of this reading disturbance. Results converge on reductions in visual processing speed for letters as the main deficit. In some dyslexic children this impairment is accompanied by reductions in *K* values, which may predict additional reading problems. In high functioning adult dyslexics, only the deficit in *C* seems to remain. Interestingly, some of the results also indicate a relation between dyslexia and *w*_index_ (leftward attentional bias), which is in line with some neuropsychological theories of dyslexia. The dyslexia studies have in several cases correlated TVA parameters with text reading speed and other markers of reading ability, which increases the clinical validity of the investigations. However, it is not clear from these investigations whether *C* or *K* is the best predictor of reading performance.

## Aging and Neurodegenerative Disorders

### Life-Span Development of TVA Parameters

Whereas normal aging is of course not a clinical condition, elderly people typically show substantial reductions of cognitive abilities compared to younger individuals. It is therefore relevant to study cognitive aging from some of the same perspectives as clinical conditions. Indeed the impairments found in studies of pathological aging, described in Section “Neurodegenerative Diseases,” can only be fully understood in the context of the normal age development. The present review therefore also includes studies that have addressed the normal life-span development of the TVA parameters.

#### McAvinue et al. (2012b): Development from 12 to 75 Years of Age

The first published study that looked into this issue was carried out by McAvinue et al. (2012b), who investigated 113 healthy participants between 12 and 75 years using the CombiTVA task. McAvinue et al. (2012b) found an approximately linear decline in the average values of *C* and *K* for each decade following the teenage years. The decline was strongest for *C*, but both parameters showed very large differences between young and elderly participants, as measured by effect sizes. The age development of *t*_0_ and α was weaker and more complex: *t*_0_ was approximately stable until the late 50s, but then increased markedly. In contrast, α increased from early adulthood until the age of about 50, but was approximately stable thereafter. The latter finding may, however, be related to the fact that α was close to the ceiling value of 1.0 in the older age groups.

#### Habekost et al. (2013): Development from 69 to 87 Years of Age

The study of McAvinue et al. (2012b) did not include participants above age 75. Habekost et al. (2013) followed up by investigating the development of whole report parameters (*C*, *t*_0_, and the visual apprehension span, an indirect measure of *K*) in a sample of 33 non-demented participants between 69 and 87 years. The results showed a marked reduction of visual processing speed with age, which was approximately halved between 70 and 85 years of age. The other two TVA parameters also declined with high age, but to a lesser extent.

#### Espeseth et al. (2014): Development from 19 to 81 Years of Age

Espeseth et al. (2014) followed up on the study by McAvinue et al. (2012b) and reported on TVA performance in 325 healthy participants between 19 and 81 years. Espeseth et al. (2014) also took the investigation further by studying how the TVA parameters relate to the integrity of the brain’s white matter, as measured by Diffusion Tensor Imaging (DTI). Espeseth et al. (2014) found that all TVA parameters expect *w*_index_ were significantly associated with age decline. As in the study of McAvinue et al. (2012b) both *C* and *K* showed an approximately linear decline from age 20 to 80, though with a weaker age effect in this study. The age effect on *t*_0_ was also similar to the previous study: early stability until the 50s, but then a marked increase toward age 80. The age development of the α parameter followed the reverse pattern: an increase in early adulthood, but relative stability in the older years. However, the mean α values were close to 1.0 already at age 40, again indicating a ceiling effect in the results. With regard to white matter integrity, Espeseth et al. (2014) found a significant relation between mean diffusivity scores and *t*_0_ values in the elderly participants. The effects were particularly seen in projection fibers such as the internal capsule, sagittal stratum, and corona radiate. This elaborates the preliminary findings on white matter lesions reported by Habekost and Rostrup (2007), and is broadly consistent with a neural interpretation of the TVA model that emphasizes the importance of thalamo-cortical projections for visual attentional computations (Bundesen et al., 2005).

#### Wiegand et al. (2014a): EEG Patterns in Young vs. Older Participants

Wiegand et al. (2014a) added another neural aspect to the understand of life-span changes in attention by showing how two EEG markers of *C* and *K*, the N1 and CDA components (see also Wiegand et al., 2014b) change significantly with aging. The anterior N1 was reduced for elderly participants with relatively low processing speed, suggesting that age-related loss of attentional resources slows encoding. Also, an enhanced right-central positivity was found for those older participants who had relatively high *K* values, pointing to additional neural recruitment for visual short-term memory in these individuals. Wiegand et al. (2014a) hypothesized that such changes in EEG components reflect cognitive processes that attempt to compensate for the capacity reductions that occur with increasing age.

#### Wilms and Nielsen (2014): Development from 60 to 75 years Age

Wilms and Nielsen (2014) used whole report testing to measure *C, K*, and *t*_0_ in a sample of 91 healthy individuals aged between 60 and 75 years. The TVA parameter estimates were compared to a range of demographic and life style variables (e.g., gender, employment status, smoking, exercise, and video gaming). A significant aging effect was found only for *C*, which surprisingly showed an improvement with age. However, this result was not significant after controlling for the influence of demographic and life-style factors. Instead the results pointed to the importance of background variables like education level, employment status, and video gaming for *C*, but not *K* and *t*_0_ (see also Nielsen and Wilms, 2015, for a structural equation modeling analysis of a related data set). The aging effects on TVA parameters found in this study, or rather lack of same, differed markedly from other findings in the field. However, as discussed by Wilms and Nielsen (2014), this may relate to biased selection and special demographic characteristics of the participant sample in this study.

### Neurodegenerative Diseases

Neurodegenerative diseases are pathological conditions which entail a progressive and debilitating loss of neurons, often developing over many years. The pathology typically starts in particular anatomical regions (e.g., the striatum or medial temporal lobe) and affects specific cognitive functions at first, but in many cases eventually spreads to other parts of the brain and causes global dementia. Prominent examples include Huntington’s and Alzheimer’s disease (AD), where TVA-based studies have so far focused, but also conditions like Parkinson’s disease and multiple sclerosis, where the first TVA-based studies have yet to appear. In recent years there has been an increasing interest in characterizing early stages of the neurodegenerative processes, as for example seen in Mild Cognitive Impairment (MCI), a precursor of AD. This has motivated an interest in using the sensitivity and specificity of TVA-based assessment to provide biomarkers for early diagnosis and to chart the general progress of the diseases.

#### Finke et al. (2006): Huntington’s Disease

The first TVA-based study on neurodegenerative diseases focused on Huntington’s disease and was carried out by Finke et al. (2006). Huntington’s disease is an autosomal dominant inherited disorder characterized by progressive degeneration of the caudate nucleus and putamen. Clinically visible symptoms of the disease typically appear in middle adulthood, but may be preceded by subtle cognitive impairments for years. Finke et al. (2006) used TVA-based testing to find cognitive biomarkers for the disease, which might aid in early identification. 18 Huntington patients were tested by whole and partial report experiments following the design of Duncan et al. (1999). The partial report experiment revealed a significant leftward bias of attentional weights (*w*_index_) in the patients, which was strongly related to age-of-onset for the first symptoms as well as to the genetic disease load (CAG repeat length). Thus the degree of lateral attentional bias seemed to mark the intensity of pathogenic mechanisms for individual patients. The finding is also consistent with the notion that the pathology in Huntington’s disease is more pronounced the left side of the brain (Rosas et al., 2001). The whole report experiment showed severe bilateral reductions of both *C* and *K*. The reductions did not correlate with age-of-onset or genetic load, but rather with the number of years since disease onset. Finke et al. (2006) therefore interpreted the two capacity parameters as biomarkers for the stage of progression in Huntington’s disease.

#### Bublak et al. (2006, 2011): Mild Cognitive Impairment and Alzheimer’s Disease

Bublak et al. (2006) suggested a generalization of the findings on Huntington’s disease to another major neurodegenerative disorder: AD, as well as a less severe condition, MCI. AD is the most prevalent neurodegenerative disease and accounts for a majority of all age-related dementia cases. Individuals with MCI are characterized by subtle cognitive impairments that lies between the normal aging pattern and dementia; a large portion of these patients develop AD within 5 years (especially those with the so-called amnestic subtype of MCI). Bublak et al. suggested that Huntington’s disease, AD, and MCI have in common a mixture of spatial and non-spatial disturbances of visual attention, which can be indexed by TVA-based assessment. This suggestion also represents an interesting parallel to the neglect syndrome, which too entails both spatial and non-spatial attention deficits. Preliminary data from 19 patients with MCI and nine patients with probable AD were mentioned by Bublak et al. (2006) to back up the hypothesis. The data indicated that, like in Huntington’s patients, visual processing speed was reduced for both AD and MCI patients. The strong leftward attentional bias of Huntington’s patients was also found in AD patients, though not in MCI patients. Thus a similar mixture of non-spatial (general) and lateralized deficits were reported across disease conditions.

The final set of results, however, diverged somewhat from this early report when they were presented by Bublak et al. (2011). In this study 18 AD patients and 18 MCI patients were tested by the whole report paradigm of Duncan et al. (1999). The results showed that deficits in MCI patients were confined to elevated visual thresholds (*t*_0_ values), whereas AD patients were also impaired in the main processing capacity parameters, *C* and *K*. The deficits in *C* and *K* also correlated with impairments on other cognitive tasks. Overall, the results showed a staged pattern of decline from MCI to AD, with deficits in visual thresholds as an early indicator of cognitive problems followed by marked capacity reductions in patients who develop AD. Interestingly, AD patients medicated with cholinesterase inhibitors had significantly better *C* values than the other AD patients (for TVA-based studies on pharmacological effects in healthy individuals, see Finke et al., 2010, and Vangkilde et al., 2011).

#### Redel et al. (2012): Mild Cognitive Impairment and Alzheimer’s Disease

Redel et al. (2012) complemented the study of Bublak et al. (2011) by employing partial report testing of patients with MCI and AD. Redel et al. (2012) used the standard paradigm of Duncan et al. to test 32 patients with MCI and 16 AD patients. Compared to a matched control group, the MCI patients showed significant impairments of both α and *w*_index_ values; further deterioration of the two parameters was observed in AD patients. Another interesting finding of this study was that individuals who were carriers of the apolipoprotein E ε4 allelle (ApoE4), a known genetic risk factor for AD, were generally characterized by a leftward spatial bias (*w*_index_). This spatial bias was also related to early disease onset. The study of Redel et al. (2012) thus presented further support for Bublak et al.’s (2006) proposal that deficits in attentional weighting are commonly associated with neurodegeneration, even at early stages of disease development.

#### Sorg et al. (2012): Mild Cognitive Development and Alzheimer’s Disease

Sorg et al. (2012) investigated the neural basis of Redel et al.’s (2012) findings on spatial bias, focusing on hypometabolism in the posterior parietal cortex, which is one of the first signs of AD development. Using a combination of positron emission tomography (PET) scans and TVA-based testing, Sorg et al. (2012) investigated the relation between this cortical hypometabolism and attentional function in seven patients with mild AD and 28 patients with prodromal AD. Prodromal AD is defined as MCI of the amnestic subtype with at least one biological sign of AD development. The participants were tested by whole and partial report following the design of Duncan et al. (1999). The theoretical interest of the study centered on *w*_index_, whereas the other TVA parameters were only used to control for other attentional subprocesses (and not discussed). The main finding of the study was that spatial bias (typically toward left-side stimuli) correlated significantly with the asymmetry of hypometabolism in the parietal cortex. The result again supports the notion that attentional deficits are an early aspect of AD development, and be detected using TVA-based testing.

### Section Summary

The studies on normal cognitive aging, with the possible exception of Wilms and Nielsen (2014), show that the two attentional capacity parameters *C* and *K* decline approximately linearly for each decade after they peak in young adulthood. The life-span development of the other TVA parameters is more complex. *t*_0_ seems to be relatively stable until the 50s, but then increases significantly in the following decades. An inverse pattern has been reported for α: decline until about age 50 followed by late stability, but the latter finding may be confounded by ceiling effects in the CombiTVA testing. *w*_index_, in contrast, seems to remain stable (i.e., symmetrical on average) across the life-span of neurologically healthy adults. The neural basis for the age-related changes in TVA parameters is also beginning to be explored. Two newly published studies point to the importance of white matter tracts for *t*_0_ and to compensatory brain activity related to *K*, respectively.

Pathological conditions associated with increasing age have been investigated in a series of TVA-based studies of neurodegenerative disorders. These studies have produced a number of interesting findings. Most importantly, they have shown how TVA based assessment can chart the progression of the diseases (from MCI to AD, and across the gradual development of Huntington’s disease). These demonstrations are generally supported by correlations between the TVA parameters and clinically relevant measures, which validates the TVA parameters as biomarkers for disease progression. Also a theoretically interesting result, which has been replicated in several studies, is the demonstration of spatial asymmetries of attentional weighting in neurodegenerative conditions that are traditionally associated with general (i.e., bilateral) cognitive decline. These findings have important perspectives for the general neuropsychological understanding of these diseases.

## ADHD and other Neurodevelopmental Disorders

In the last few years a fourth research area has emerged for clinical TVA-based investigations: studies of neurodevelopmental disorders. Dyslexia, which has already been treated in the context of reading disturbances in Section “Dyslexia”, may be regarded as one such condition. Other examples are preterm birth and congenital brain malformations (see Other Neurodevelopmental Conditions). At present most research within this field, both published and on-going, however, seems to be directed at the syndrome of Attention deficit hyperactivity disorder (ADHD).

### Attention Deficit Hyperactivity Disorder

Attention deficit hyperactivity disorder is a highly prevalent psychiatric disorder that is characterized by symptoms of inattention, impulsivity, and hyperactivity. ADHD symptoms emerge in childhood and in many cases persist into adulthood. The core cognitive deficit (or deficits) underlying the behavioral manifestations of ADHD is much debated, with suggestions including deficient working memory, delay aversion, and hypoarousal (Castellanos et al., 2006). Several of these hypotheses are directly translatable into TVA terms: for example, a deficit in working memory may correspond to *K* reduction, whereas hypoarousal can be related to lower *C* values (see Bundesen et al., 2015, for a theoretical account of the relation between visual processing speed and arousal). Based on such hypotheses, TVA-based studies attempt to reveal the core deficits in ADHD.

#### Finke et al. (2011): Adult ADHD

The first study was made by Finke et al. (2011), who investigated a group of 30 adults with ADHD. The group was carefully screened for comorbidities, which are otherwise very frequently occurring in persons with ADHD. Finke et al. (2011) tested the participants using the standard whole and partial report paradigms of Duncan et al. (1999). They found a selective deficit in the *K* parameter (with medium to high effect size), whereas *C*, *w*_index,_ and α values were not significantly different from the control group. The *K* values did not correlate significantly with age, IQ, income, or other socio–economic measures and thus seemed to represent a relatively pure neurocognitive endophenotype for ADHD. The results thus support theories of deficient working memory as the primary deficit in ADHD.

#### McAvinue et al. (2012a): Childhood ADHD

Quite different results were obtained by McAvinue et al. (2012a), who studied 25 children (9–13 years) with ADHD using the CombiTVA paradigm. In contrast to Finke et al. (2011), McAvinue et al. (2012a) found selective deficits in the *C* parameter, whereas *K* and the other standard TVA parameters did not differ significantly from the control group. Notably, McAvinue et al. (2012a) fitted the data by a new algorithm that included estimation of the frequency of attentional lapses (see Developments in Data Analysis). Lapses represent trials in which participants are off-task (i.e., reporting zero items in spite of a relatively long exposure time). The lapse frequencies of children with ADHD were even more different from the control group (i.e., the effect size was larger) than was the case for visual processing speed. Overall, the results of McAvinue et al. (2012a) seem to favor an arousal-related model of ADHD, which can explain the slower processing and the frequent lapses of attention.

### Other Neurodevelopmental Conditions

#### Caspersen and Habekost (2013): Spina Bifida Myelomeningocele

The heterogeneity that characterizes many neurodevelopmental conditions was also evident in a study by Caspersen and Habekost (2013). Caspersen and Habekost (2013) studied six children with spina bifida myelomeningocele (SBM), a congenital defect in the neural tube which often leads to cerebral disturbance. This is the first TVA-based study of a neuropaediatric disorder. On a group level, the children with SBM had significantly poorer α values than the control group. However, the most informative results were demonstrated at single case level. Each of the six children deviated significantly from the control group on one or more test variables (e.g., *t*_0_, *C*_left_, or *K*). The individual patterns of deficits were quite different even though all children shared the same general neurological condition. Thus the study showed the usefulness of TVA based assessment to provide individual deficit profiles, and demonstrated the heterogeneity of cognitive deficits after SBM.

#### Finke et al. (2015): Preterm Born Adults

Finke et al. (2015) used whole and partial report experiments to study another population with developmental disturbances: adults who are born preterm. 33 preterm individuals were compared to 32 full-term born participants. Resting state fMRI was also included in the investigation to obtain measures of functional connectivity of intrinsic brain networks relevant for visual attention. Finke et al. (2015) found a selective deficit in the *K* parameter in the preterm group, while the other parameters did not differ from the control group. Among preterm born adults, individual patterns of changed connectivity in occipital and parietal cortices were systematically associated with visual short-term memory function in such a way that the more distinct the connectivity differences, the better the preterm adults’ *K* score. Finke et al. (2015) suggested that the changes reflect processes that attempt to compensate for the adverse developmental consequences of prematurity.

### Section Summary

Compared to the other research areas reviewed in this article, the studies on neurodevelopmental conditions have produced rather divergent results. The complexity of the findings is partly related to the fact that quite different clinical conditions have been studied. However, findings within the same patient group have also differed substantially. This is perhaps not surprising, given the heterogeneity of many diagnostic categories in neuropsychiatry and neuropaediatrics (e.g., SBM). Still it is important to consider the theoretical implications of the divergent findings, especially for ADHD, where several well-defined hypotheses have been tested. In particular, it is not presently clear how to reconcile the opposite findings on *K* and *C* from the two studies on children and adults with ADHD. One possibility is that adults with ADHD represent a special subtype (“persisters”) of the general population of individuals who develop ADHD. A different, methodological explanation is that the individuals studied by Finke et al. (2011) represent a more pure sample of ADHD compared to the sample of McAvinue et al. (2012a), which was not similarly screened for comorbidities or other confounding factors. It is also possible that the divergent findings are related to the use of different variants of TVA based assessment (CombiTVA vs. the Duncan et al.-paradigm) but it is not clear why the former paradigm should be more sensitive to *C* deficits and the latter to deficits in *K*. Hopefully, future studies on ADHD will provide more clarity on this issue.

## General Discussion

See **Table 1** for an overview of the clinical TVA-based studies that have been published until now, categorized under the four research areas presented in this article. Besides the clinical condition and the number of patients investigated, the table lists the central findings of each study in terms of which TVA parameters were affected. As described in Sections “Neglect and Simultanagnosia” to “ADHD and other Neurodevelopmental Disorders”, the individual findings are often complex and require a detailed understanding of each study in order to be interpreted. However, one thing is immediately evident from the table: there is a high degree of overlap between the affected TVA parameters across research fields. In particular, reductions in both *C* and *K* are found in such diverse groups as patients with neglect, pure alexia, Huntington’s disease, and ADHD. This has important implications for how one should understand the specificity of TVA-based assessment. The assessment is clearly not clinically specific in the sense that deficits in particular parameters are diagnostic of particular conditions. Basic diagnosis will have to be carried out by other means. Instead, the specificity of the assessment method is cognitive: it can point to impairments in well-defined cognitive functions, deficits that may be shared with other clinical conditions, but are functionally distinct from other types of attentional deficits. The clinical value of TVA-based assessment thus lies primarily in its ability to elaborate on the basic diagnosis and provide a detailed characteristic of the pattern of attentional difficulties for a given individual or group of patients.

**Table 1:** Clinical theory of visual attention (TVA)-based studies.

	Clinical condition	Patient sample size	Main findings
**Neglect and simultanagnosia**
Duncan et al. (1999)	Neglect	9	*K*, *C, w*_index_, *α, A*
Habekost and Bundesen (2003)	Subclinical neglect	1	*K, w*_index_, *t*_0left_
Habekost and Rostrup (2006)	Right hemisphere stroke	26	*K, C*_left_, *w*_index_
Habekost and Rostrup (2007)	Right hemisphere stroke	22	*K*, *C*_right_
Kraft et al. (2015)	Thalamic stroke	16	*C, w*_index_
Peers et al. (2005)	Parietal vs. frontal strokes	25	*K, C,*α
Bublak et al. (2005)	Parietal vs. frontal stroke	2	*w*_index_, α, *A*
Finke et al. (2012)	Phasic alerting of neglect	6	*w*_index_, *A*
Duncan et al. (2003)	Dorsal simultanagnosia	1	*C*
Finke et al. (2007)	Simultanagnosia/Huntington’s disease	10	*C*
***Reading disturbances***
Duncan et al. (2003)	Ventral simultanagnosia	1	*C, w*_index_
Starrfelt et al. (2009)	Pure alexia	4	*C*_letters_, *C*_digits_*, K*_letters_, *K*_dig_
Starrfelt et al. (2010)	Pure alexia	1	*C*_letters_, *C*_digits_*, K*_letters_, *K*_dig_
Habekost et al. (2014a)	Pure alexia	4	*C*_letters_, *C*_words_
Habekost and Starrfelt (2006)	Quadrantamplyopia	1	*C*_upperrightquadrant_
Dubois et al. (2010)	Childhood dyslexia	2	*K, C*
Bogon et al. (2014)	Childhood dyslexia	12	*K, C*
Stenneken et al. (2011)	Adult dyslexia	23	*C*
***Aging and neurodegenerative disorders***
McAvinue et al. (2012b)	Healthy, 12–75 years	(113)	*K, C,*α*, t_0_*
Habekost et al. (2013)	Non-demented, 69–87 years	(33)	*K, C, t_0_*
Wiegand et al. (2014a)	Healthy, young vs. elderly	(40)	*K, C*
Espeseth et al. (2014)	Healthy, 19–81 years	(325)	*K, C,*α*, t_0_*
Wilms and Nielsen (2014)	Healthy, 60–75 years	(91)	*K, C, t_0_*
Finke et al. (2006)	Huntington’s disease	18	*K*, *C, w*_index_
Bublak et al. (2006)	Mild Cognitive Impairment and probable Alzheimer’s disease (AD)	28	*C, w*_index_
Bublak et al. (2011)	Mild Cognitive Impairment and AD	36	*K, C, t_0_*
Redel et al. (2012)	Mild Cognitive Impairment and AD	48	α, *w*_index_
Sorg et al. (2012)	Mild and prodromal AD	35	*w*_index_
**Neurodevelopmental disorders**
Finke et al. (2011)	Adult ADHD	30	*K*
McAvinue et al. (2012a)	Childhood ADHD	25	*C*, *lapse*
Caspersen and Habekost (2013)	Spina bifida myelomeningocele	6	*K*, *C*_left_*,*α, *t*_0_
Finke et al. (2015)	Preterm born adults	33	*K*

The cognitive specificity of TVA-based assessment seems equally well suited for studying effects of focal brain lesions as well as clinical conditions with diffusely distributed pathology affecting large-scale brain networks (e.g., neurodegenerative diseases). Especially in the latter type of conditions, the ability to characterize attentional impairments by a well-defined parameter profile goes well beyond conventional clinical assessment approaches. Thus, TVA-based assessment has great potential not only for improving the understanding of generalized brain diseases but also for establishing neuropsychological measures as valid biomarkers.

This perspective of cognitive profiling makes it important to understand the wider significance of each TVA parameter, including its relation to other cognitive and neuropsychological constructs. Starting with visual processing speed *C*, this parameter provides a measure of the efficiency of visual form recognition (for a given stimulus type). There are many other measures of processing speed in the neuropsychological literature, but most of them are reaction-time based (e.g., the Alertness task in the TAP battery, Zimmermann and Fimm, 1993) and therefore not specific to processing in the visual system. Other accuracy-based measures of visual processing speed do exist, for example the useful field of view (Ball and Owsley, 1993) and the inspection time (Deary and Stough, 1996). Like TVA-based assessment these tests use very brief visual presentations. However, these testing conditions mean that performance can be strongly influenced by individual differences in perception thresholds as well as in visual processing speed. TVA-based analysis is currently the only way to distinguish between these two factors. Concerning the clinical significance of visual processing speed, the frequent findings of *C* reductions across neuropsychological conditions suggest that the parameter is vulnerable to disturbance in many different brain regions, also outside cortical visual areas (see Habekost and Starrfelt, 2009, for a detailed discussion on the lesion anatomy of *C* and *K*). Besides the parameter’s specific relation to visual form recognition abilities, *C* can therefore be seen as a sensitive marker for the general processing efficiency of the brain.

The other main parameter of attentional capacity, *K*, represents the maximum number of visual objects that can be perceived simultaneously. As such, it is related to other measures of visuo-spatial working memory. For example, Finke et al. (2005) found a moderately significant correlation between *K* and the Visual Memory Span from the WMS-R battery (Härting et al., 2000). However the *K* parameter is arguably a very pure estimate of visual apprehension span, both due to the minor response requirements of the whole report task as well as the power of TVA analysis, which controls the *K* estimate for the influence of other visual factors (e.g., processing speed). One objection to this latter argument is that *C* and *K* typically correlate significantly, which may question their separability. However, correlation need not contradict independence; weight and height are also correlated, but clearly represent distinct aspects of bodily structure. *K* and *C* are estimated mathematically independently and, given data of sufficient quality (i.e., several hundred trials and performance spanning from threshold to ceiling), it should be possible to distinguish reliably between the two parameters even though they tend to co-vary in the same individuals. In terms of clinical significance, *K* has been found impaired in many different conditions (e.g., neglect, ADHD, or Huntington’s disease). Like for *C*, there are many theoretical and empirical reasons to assume that *K* depends on large anatomical networks that involve many parts of the brain including white-matter connectivity (Habekost and Rostrup, 2007; Habekost and Starrfelt, 2009). Also the *K* parameter can therefore be taken as an indicator of the brain’s general processing efficiency, besides its specific relation to simultaneous visual perception.

Parameter α represents the effectiveness of top–down control of visual attention. Also for this parameter analogous cognitive measures exist. For example, Finke et al. (2005) compared α to performance on a Stroop task and found a moderate correlation. A similar correlation was found to the executive control network parameter of the ANT task (Habekost et al., 2014b). The fact that these correlations were only modest is probably due to the fact that both alternative measures of attentional control (as well as most other tests in the literature) also depend strongly on motor-related processes like response inhibition. In contrast, the partial report task specifically assesses perceptual processes in attentional control (i.e., visual filtering). Clinically, findings on α deficits have been surprisingly sparse and are limited to a few studies (Bublak et al., 2005; Peers et al., 2005; Redel et al., 2012). There may be several explanations for this. On the methodological side, estimation of α is generally less reliable than the other TVA parameters, and there may be ceiling effects in the CombiTVA testing of this parameter. However α still has a reliability level that is comparable to many other neuropsychological measures (Habekost et al., 2014b) and the Duncan et al. paradigm is not characterized by ceiling effects in α. It is therefore likely that top–down selectivity in visual attention is simply more robust to many kinds of brain disturbances than the other TVA parameters, and perhaps mainly vulnerable to very large lesions (Peers et al., 2005) or damage to the superior frontal lobe (Bublak et al., 2005). The latter type of brain disturbance is relatively rare after stroke, but may be more common in AD even at early stages (Redel et al., 2012).

Parameter *w*_index_ is a measure of lateral attentional bias in visual perception. In the cognitive literature it is conceptually associated with measures like spatial orienting (although *w*_index_ did not correlate with this parameter in the ANT test; Habekost et al., 2014b) and the Visual Scanning subtest of the TAP battery (where Finke et al., 2005, did find a significant correlation). Clinically, *w*_index_ is most directly associated with the phenomenon of visual extinction (i.e., competition for conscious perception between bilaterally presented stimuli; Bender, 1952). Compared to such alternative cognitive or clinical measures of spatial bias, the estimation of *w*_index_ has the same advantages as the other TVA parameters: it is controlled for the influence of confounding motor and visual factors, particularly side differences in sensory effectiveness. Further, the findings across the clinical TVA literature suggests that *w*_index_ is a sensitive indicator of brain asymmetry, whether it be caused by unilateral stroke or asymmetrical neurodegeneration. Besides the typically strong effects of large unilateral brain damage on *w*_index_, a theoretically interesting finding is the relation between *w*_index_ and lesions in the thalamic pulvinar nucleus (Habekost and Rostrup, 2006; Kraft et al., 2015), which is consistent with the neural TVA model of Bundesen et al. (2005).

The fifth TVA parameter, *t*_0_, represents the lower temporal threshold for visual perception. The parameter is related to traditional psychophysical measures of perceptual thresholds. *t*_0_ has generally received less interest than the other four TVA parameters and is sometimes treated merely as a control variable for valid *C* estimation. However, recent findings indicate that changes in *t*_0_ may be an important marker for pathological aging, as seen in MCI (Bublak et al., 2011) or changes in white matter connectivity (Espeseth et al., 2014). Future studies will clarify whether the *t*_0_ parameter deserves more attention that it has received hitherto.

## Future Directions

Clinical TVA-based studies can be predicted to go in several directions in the coming years. One development may be a further widening of the clinical scope for the studies. Over the last 15 years TVA-based assessment has been applied to an increasing number of clinical conditions, as documented in this review. However, attentional disturbances are a significant part of most neurological and psychiatric conditions, so there still seems to be room for many novel investigations. For example, currently on-going studies include patients with multiple sclerosis, Tourette’s syndrome, congenital prosopagnosia, and traumatic brain injury. Other relevant topics for TVA-based studies that still await investigation are Parkinson’s disease or schizophrenia. Also, given that many questions still remain for the clinical conditions that have been studied previously by TVA methods (e.g,. ADHD or dyslexia), studies are likely to continue within the already established research areas.

A second main development in the coming years might be a stronger combination of clinical TVA-based assessment with treatment programes and supplementary clinical and neuroimaging measures. As noted in the introduction, a number of studies have investigated the effects of cognitive or physiological interventions on TVA parameters in healthy participants. Given that several of these interventions (e.g., cognitive training or pharmacological substances) have large relevance for clinical treatment, it seems promising to design studies where the clinical effects of such interventions are monitored by TVA-based assessment. To further strengthen the clinical relevance of such investigations, it would also be natural to include a wider range of clinical and biological measures on the patients. This can be seen as a continuation of the biomarker approach taken in many of the studies on neurodegenerative diseases, but broadening the investigations further to include more information on for example genetic properties or neurotransmitter levels. Finally, given Wiegand et al.’s promising findings on EEG markers for *C* and *K*, such measures of brain function is also likely to grow in importance for clinical TVA-based research in the coming years.

## Conclusion

Since the first article was published in Duncan et al. (1999), about 30 studies have used TVA-based assessment to investigate attentional deficits in various neurological and psychiatric conditions. Clinical TVA-based studies have so far focused on four main research areas: (1) neglect and related conditions, (2) reading disturbances, (3) aging and neurodegenerative diseases, and (4) neurodevelopmental disorders. Typically the main aim of these studies has been to use the specificity of TVA-based assessment to address theoretical hypotheses about the core deficits of the disorders. The findings are generally consistent across studies and have often added substantially to the theoretical understanding of the conditions, although decisive results on the core deficits of, for example, pure alexia and ADHD still remain elusive. The clinical validity of the assessment has been supported by some studies, especially on neurodegenerative diseases, which have related the TVA parameters to clinically relevant behavior or biological disease markers. However, in other fields of TVA-based research, the relation to other clinical measures needs to be further established.

The main strength of TVA-based assessment method lies in its theoretical grounding and cognitive specificity: the ability to measure five theoretically central aspects of visual attention. As additional qualities the method has also proven sensitive to minor attentional disturbances, shown good reliability, and can be adapted to many different types of clinical investigations. Looking toward future studies, the list of neuropsychological conditions that can be meaningfully addressed by TVA based assessment is far from exhausted, and the next in line may be multiple sclerosis, Tourette’s syndrome, and traumatic brain injury. Besides studying new patient populations, it also seems promising to combine TVA-based assessment with cognitive and pharmacological interventions, and to include biological disease markers and EEG measures in the investigations.

## Conflict of Interest Statement

The author declares that the research was conducted in the absence of any commercial or financial relationships that could be construed as a potential conflict of interest.
